# The efficacy and safety of tacrolimus on top of glucocorticoids in the management of IIM-ILD: A retrospective and prospective study

**DOI:** 10.3389/fimmu.2022.978429

**Published:** 2022-09-02

**Authors:** Yuxue Chen, Zhiqian Bai, Ziyun Zhang, Qiongjie Hu, Jixin Zhong, Lingli Dong

**Affiliations:** ^1^ Department of Rheumatology and Immunology, Tongji Hospital, Tongji Medical College, Huazhong University of Science and Technology, Wuhan, China; ^2^ Department of Radiology, Tongji Hospital, Tongji Medical College, Huazhong University of Science and Technology, Wuhan, China

**Keywords:** idiopathic inflammatory myopathies (IIM), interstitial lung diseases (ILD), glucocorticoids, tacrolimus, pirfenidone

## Abstract

**Objective:**

To examine the efficacy of tacrolimus on top of glucocorticoids (GCs) in the management of idiopathic inflammatory myopathies-associated interstitial lung disease (IIM-ILD) and further assess the therapeutic benefit and safety of low-dose pirfenidone followed above treatments.

**Methods:**

The retrospective study comprised 250 patients with IIM-ILD hospitalized in Tongji Hospital from 2014 to 2020. Demographic data, survival outcomes, and recurrence rates over the 1-year follow-up period were retrospectively analyzed. These patients were divided into two groups based on treatment with tacrolimus alone or other conventional immunosuppressants. Endpoints were compared by adjusted Cox regression model using inverse probability of treatment weighting to minimize treatment bias and potential confounders. For the prospective study, IIM-ILD patients treated with tacrolimus alone or tacrolimus combined with low-dose pirfenidone were enrolled from 2018 to 2020. Clinical characteristics, survival outcomes and multifarious assessment scales were followed up at baseline, 3, 6 and 12 months. The primary endpoint was 12-month survival rate and the secondary endpoints included respiratory-related events, adverse events, exacerbation in HRCT findings and laboratory parameters during therapy courses, and changes in respiratory function.

**Results:**

For the retrospective study, tacrolimus group (n=93) had a significantly higher survival rate (weighted HR=0.330, p=0.002) and a lower relapse rate (weighted HR=0.548, p=0.003) compared with patients treated with other types of immunosuppressant (n=157) after adjustment. The prospectively enrolled 34 IIM-ILD patients were treated with tacrolimus (n=12) or tacrolimus combined with low-dose pirfenidone (n=22). After 12 months of treatment with tacrolimus, patients in the prospective cohort showed significant improvements in cardio-pulmonary function, disease activity, muscle strength, and mental scale from baseline. Subgroup analysis indicated that patients with tacrolimus and pirfenidone combination therapy showed lower chest HRCT scores (p=0.021) and lower respiratory-related relapse rates than those in tacrolimus monotherapy group (log-rank p=0.0029). The incidence rate of drug-associated adverse events (AEs) was comparable between two groups and none of the patients discontinued the treatment due to severe AEs.

**Conclusion:**

Tacrolimus is well-tolerated and effective in the treatment of IIM-ILD. Furthermore, low-dose pirfenidone add-on treatment seems result in favorable improvements in pulmonary involvements for IIM-ILD patients.

**Clinical Trial Registration:**

http://www.chictr.org.cn, identifier ChiCTR2100043595.

## Introduction

Idiopathic inflammatory myopathy (IIM) is a group of heterogeneous autoimmune disorders characterized by muscle weakness and non-suppurative inflammation of skeletal musculature. A range of extra-muscular organs may also be involved, such as heart, lung, and joints. Among those organ involvements, interstitial lung disease (ILD) is the most common pulmonary complication with high morbidity and mortality, which is one of the dominant factors responsible for the poor prognosis of these patients (1). There are no standard guidelines for the treatment of IIM-associated ILD (IIM-ILD). Glucocorticoids (GCs) combined with immunosuppressive agents including methotrexate (MTX), cyclophosphamide (CTX), mycophenolate mofetil (MMF) or azathioprine (AZA) are widely used as the conventional clinical strategy (2). However, the therapeutic effects are controversial and a substantial proportion of patients, especially those with rapidly progressive ILD (RPILD), respond poorly to this regimen (3).

Tacrolimus is an immunosuppressant that acts as a calcineurin inhibitor and selectively suppresses T lymphocytes proliferation and interleukin-2 transcription (4). It also suppresses collagen synthesis and expression of the transforming growth factor beta 1 (TGF-β1) receptor in lung fibroblasts (5). Based on these findings, tacrolimus seems to be a promising agent for IIM-ILD patients. Recently, the efficacy of tacrolimus acting as an add-on therapy in connective tissue disease-associated ILD has been demonstrated in several case reports, retrospective studies and some clinical trials (6–11). Pirfenidone (5-methyl-1-phenyl-2-[1H]-pyridone), an oral bioavailable synthetic agent with tolerable adverse effects, has been approved for the treatment of idiopathic pulmonary fibrosis (IPF). Pirfenidone could play anti-inflammatory and anti-fibrotic effects through down-regulating a series of cytokines, including TGF-β1, tumor necrosis factor alpha (TNF-α), connective tissue growth factor (CTGF), and platelet-derived growth factors (PDGF) (12, 13). It is reported that pirfenidone could ameliorate the decline of pulmonary function and improve progression-free survival in IPF patients (14–16). Meanwhile, Li T et al. noted that pirfenidone, as an add-on therapy, may improve the prognosis of patients with subacute ILD related to clinical amyopathic dermatomyositis (17). But full dose of pirfenidone have heavy gastrointestinal and financial burden, which may reduce medical order compliance. Up to now, the efficacy of low-dose pirfenidone combined with tacrolimus and GCs in patients with IIM-ILD has not been reported.

In the present study, we aim to investigate the efficacy of tacrolimus on top of GCs in management of IIM-ILD in a retrospective analysis. Then we conducted a prospective study to further evaluate the value of tacrolimus and assess the efficacy and tolerability of low-dose pirfenidone based on the above treatments in IIM-ILD patients.

## Materials and methods

### Study design

A retrospective cohort study followed by a prospective cohort study were conducted in Tongji Hospital, Wuhan, China. We enrolled patients diagnosed with IIM according to the criteria of Bohan and Peter (18, 19) or CADM by Sontheimer et al. (20, 21). The diagnosis of ILD was in accordance with the respiratory symptoms, physical examinations, typical high-resolution computed tomography (HRCT) findings, and restricted dyspnea detected by pulmonary function tests. Patients with inclusion body myositis, malignancy-associated or overlapping myositis were excluded. The study was approved by Institutional Review Board of Tongji Medical College, Huazhong University of Science and Technology in accordance with the principles of the Declaration of Helsinki and registered online at the Chinese clinical trial (ChiCTR2100043595).

### Retrospective observational group patients

The medical records of IIM-ILD admitted to Department of Rheumatology and Immunology of Tongji Hospital from January 2014 to September 2020 were retrospectively retrieved. For the retrospective study, patients were divided into two groups according to their treatments. The conventional therapy group was defined as the patients treated with GCs combined with any other immunosuppressive agents except for tacrolimus, while the tacrolimus group was defined as the patients treated with GCs in combination with tacrolimus and was not exposed to any other immunosuppressants besides glucocorticoids.

The total follow-up duration was 12 months. The primary endpoint was 12-month survival rate and the secondary endpoint was the time from initiation of treatment to relapse. Relapse was defined as when patients appeared exacerbation of symptoms combined with one of the following conditions: i) increase in the level of serological parameters to >2-fold greater than the baseline level, such as ferritin and erythrocyte sedimentation rate (ESR); ii) radiological progression of ILD evaluated by both rheumatologists and radiologists; iii) requirement for treatment of increased dose of glucocorticoids (>0.5 mg/kg/day). Respiratory-related relapse was defined as the exacerbation of respiratory-related symptoms combined with radiological progression of ILD evaluated by both rheumatologists and radiologists. Written informed consent was waived due to the retrospective nature of the study design. In order to minimize the bias in assessment of observational cohort data, we utilized the inverse probability of treatment weighting (IPTW) method to eliminate any difference between the two groups to evaluate the efficacy of tacrolimus in the treatment of IIM-ILD

### Prospective investigation group patients

In the prospective cohort study, we enrolled patients who were diagnosed with IIM-ILD from 2018 to 2020, and then treatment of GCs and tacrolimus was initiated. The exclusion criteria included: accompanied with severe respiratory-related disease, cancer, active tuberculosis, severe immune-deficiency disorders, renal inefficiency, and patients under 18 years old. GCs was initially administered at 0.8-1.5 mg/kg/day of prednisolone or its equivalent for 4 weeks, thereafter, the existing dose was reduced by 5 mg/day of prednisolone or its equivalent every 4 weeks when the dose was above 20 mg daily. When the daily dosage was below 20mg, the dose was reduced by 2.5 mg/day of prednisolone or its equivalent every 2-4 weeks. The use of GCs should be kept at the lowest possible dose. Oral tacrolimus was given twice daily (0.075 mg/kg of body weight) to achieve a plasma trough level of 5–10 ng/ml. The dosage was subsequently adjusted based on response to therapy and findings of toxicity monitoring. Patients who additionally received pirfenidone entered into combination group. Low dose of pirfenidone was defined as 600-900 mg/day and continued for 12 months. Additional therapies such as plasma exchange and intravenous immunoglobulins were permitted when the patient’s condition worsened.

The follow-up period was 12 months. Study visits were set at baseline, 1, 3, 6, and 12 months after the start of the treatment, and until the end of the 12 month or the time of study withdrawal. The primary endpoint was 12-month survival rate. The secondary endpoints included the following: respiratory-related events, adverse events, exacerbation in HRCT findings and laboratory parameters during therapy courses, and changes in respiratory function. Adverse events associated with the treatment protocol were recorded during the observational period. Written informed consent was obtained from all patients before participating.

### Clinical and laboratory examinations

Clinical features including age, gender, smoking history, disease duration, diagnosis and comorbidity, skin rashes as well as concomitant therapy modalities were recorded. Laboratory tests including blood counts, hepatic and renal functions, immunological examinations, myositis related specific antibodies, C-reactive protein (CRP), ESR, creatine kinase (CK) and serum ferritin levels were conducted at every medical visit.

### Myositis assessment scales

For the prospective group, we compared the treatment efficacy between subgroups *via* several Myositis Assessment Scales according to previous report (22). The measurement tools applied in our study included: 6-min walking test (6MWT), modified Medical Research Council (mMRC), Patient Global Activity, Manual Muscle Testing (MMT) -8, Myositis Disease Activity Assessment visual analog scale (MYOACT), Myositis Intention to Treat Activities Index (MITAX), Myositis Damage Index (MDI), Cutaneous Assessment Tool (CAT), and Hamilton Anxiety Scale (HAMA), and Hamilton Depression Scale (HAMD).

### Interpretation of chest HRCT scans and scores

In the prospective study, baseline and the final chest HRCT scans were independently evaluated by two professional radiologists who were blinded for grouping information according to previous methods (23, 24). Cases who died during observational period were not included into the analysis. First, the radiologists classified HRCT findings into the following clinical phenotypes: non-specific interstitial pneumonia (NSIP), usual interstitial pneumonia (UIP), organizing pneumonia (OP), NSIP with OP, acute interstitial pneumonitis (AIP), or unclassifiable pattern. Subsequently, the radiologists scored the chest HRCT abnormalities including ground-glass attenuation, airspace consolidation, interlobular septal thickening and/or reticular opacity and traction bronchiectasis in each of lobes from right and left lungs based on a semi-quantitative assessment. The total HRCT score was calculated as the sum of the scoring of each lobe fibrosis lesions. Scoring for the extent of pulmonary fibrosis in each lung lobe was graded on a scale of 1 to 4 as follows: area with 1 = 0–25%, 2 = 26–50%, 3 = 51–75%, and 4 = 76–100%.

### Statistical analysis

Continuous variables are shown as mean ± standard deviation or median and interquartile range as appropriate. The differences between subgroups were compared with the Mann-Whitney U test or Student’s t-test for continuous variables and the Chi-squared or Fisher’s exact test for categorical data. Changes in clinical assessment indicators between baseline and each visit point were analyzed using the Wilcoxon t-test. The Bonferroni post-test correction was used to reduce the likelihood of false positives. Survival curves were conducted by Kaplan-Meier methods and differences between subgroups were compared with a log-rank test. Analysis of primary and secondary endpoints were compared between subgroups was based on adjusted Cox regression model using IPTW (25).

The propensity score is defined as a patient’s probability of treatment selection (tacrolimus use), conditional on observed baseline variables. Subjects weighted by the inverse probability of therapeutic modality received generates a synthetic sample in which treatment assignment is not dependent of observed baseline covariates. IPTW by using the propensity score allows each patient to obtain unbiased estimates of average treatment efficacies. In this present study, the propensity score was calculated for each subject using a logistic regression model including age, sex, the diagnosis, initial Prednisolone dose, plasma exchange (PE) and intravenous immunoglobulin (IVIg), which were selected as possible confounders to enter into logistic regression model.

The probability of each patient receiving treatment regimen was calculated based on propensity score methods. The weight for patient in tacrolimus group was the inverse of propensity score and the weight for patient from the control group was calculated as 1/(1-propensity score). The Kaplan-Meier curves were illustrated and the adjusted HR and a robust 95% CI were estimated in the weighted sample using a Cox regression model.

All the data was analyzed using R (version 4.1.0; R Foundation for Statistical Computing, Vienna, Austria), IBM the SPSS version 25.0 (SPSS Inc., Chicago, IL, USA) and Graphpad Prism version 8.4 (GraphPad Software, San Diego, CA, USA). A two-tailed p value<0.05 was considered as statistically significant.

## Results

### Results of retrospective cohort study

**Figure 1:**
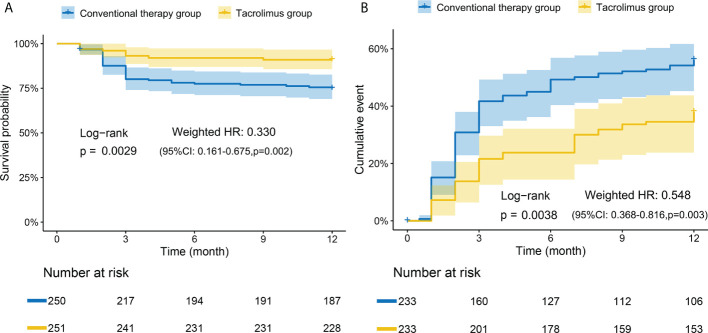
Kaplan-Meier curves of tacrolimus group and conventional therapy group. **(A)** Adjusted survival rate curves. Tacrolimus group had a significantly higher overall survival rate than conventional therapy group. **(B)** Adjusted relapse rate curves. The conventional therapy group had a significantly higher relapse rate than tacrolimus group. Adjusted for: age, gender, diagnosis subsets, initial glucocorticoids doses, plasma exchange (PE), intravenous immunoglobulin (IVIg). The numbers at the bottom of the chart represent the weighted population distribution between the two groups for different observation time points.

A total of 250 patients consisting of 93 patients treated with tacrolimus and 157 patients received other conventional therapies were consecutively enrolled in the retrospective study (**Supplementary Figure S1**). There was no significant difference in clinical characteristics including age, gender, disease duration, and diagnosis subsets between the two groups (**Table 1**). In the conventional therapy group, cyclophosphamide (CTX) was the most frequently used immunosuppressive agent, followed by methotrexate (MTX) and azathioprine (AZA). The survival curves of the two groups were weighted by using IPTW. A significant improvement in 12-month survival rate after adjustment was observed in tacrolimus group compared to conventional treatment group after adjustment (log-rank p=0.0029, weighted HR=0.330, 95% CI: 0.161-0.675, P=0.002) (**Figure 1A**). 39 patients (38.7%) and 81 patients (51.6%) experienced relapse events within one years in tacrolimus group and conventional therapy group, respectively. After adjustment, the tacrolimus group showed a significantly lower relapse rate compared with the conventional therapy group (log-rank p=0.0038, weighted HR=0.548, 95% CI: 0.368-0.816, P=0.003) (**Figure 1B**). The outcomes of several other common immunosuppressive agents including CTX, MTX and AZA were compared with the tacrolimus group separately. As shown in **Supplementary Figure S2**, the 12-month survival rate was significantly improved by tacrolimus when compared with AZA. The relapse-events rates were significantly lower in tacrolimus group compared to MTX or CTX group.

**Table 1:** Clinical characteristics of patients of retrospective cohort.

Characteristics	Tacrolimus group (n=93)	Conventional therapy group (n=157)	P value
Age (years), mean (S.D.)	51.65 (11.27)	51.90 (10.74)	0.986
Female sex, no (%)	54 (70.0)	103 (65.6)	0.485
Diagnosis, no. (%)			
DM	60 (64.5)	112 (71.3)	0.484
PM	22 (23.7)	32 (20.4)	
CADM	11 (11.8)	13 (8.3)	
Therapy			
Initial Prednisolone dose (mg/day)	54 (24)	53 (29)	0.732
IVIg, no. (%)	21 (13.4)	17 (18.3)	0.297
Plasma exchange, no. (%)	17 (10.8)	10 (18.3)	0.097
Tacrolimus, no. (%)	93 (100.0)	0	–
CTX, no. (%)	0	69 (43.9)	
MTX, no. (%)	0	51 (32.5)	
AZA, no. (%)	0	15 (9.6)	
LEF, no. (%)	0	9 (5.7)	
CsA, no. (%)	0	9 (5.7)	
MMF, no. (%)	0	9 (5.7)	
Tripterygium glycosides, no. (%)	0	30 (19.1)	

S.D, standard deviation; DM, dermatomyositis; PM, polymyositis; CADM, clinical asympmyopathic dermatomyositis; IVIg, intravenous immunoglobulin; CTX, cyclophosphamide; MTX, methotrexate; AZA, azathioprine; LEF, leflunomide; CsA, ciclosporin; MMF, mycophenolate.

### Results of prospective cohort study

Forty-one patients were prospectively enrolled, and seven patients were excluded from the prospective cohort due to the loss of follow-up (n=2), premature discontinuation of intervention (n=2) and other reasons (n=3). 22 patients and 12 patients were included in the combination therapy group and tacrolimus group, respectively (**Supplementary Figure S3**). Baseline clinical characteristics of both groups were displayed in **Table 2**. There were no significant differences in demographic data, positivity rate of anti-MDA-5-antibody, laboratory indicators, HRCT findings, and concomitant therapy modalities between two groups.

**Table 2:** Clinical characteristics of the patients of prospective cohort at the time of enrollment.

Characteristics	Tacrolimus group (n=12)	Tacrolimus + Pirfenidone group (n=22)	P value
Age (years), mean (S.D.)	52.8 (11.2)	50.0 (10.3)	0.435
Female sex, no (%)	6 (50.0)	14 (63.6)	0.440
Disease duration (months), median (IQR)	3 (1-10)	3 (1-7)	0.844
Smoke history, no (%)	3 (25.5)	5 (22.7)	0.881
Diagnosis, no. (%)			
DM	5 (41.7)	12 (54.5)	0.597
PM	3 (25.0)	6 (27.3)	
CADM	4 (33.3)	4 (18.2)	
Heliotrope rash, no. (%)	4 (33.3)	13 (59.1)	0.151
Gottron’s sign, no. (%)	6 (50.0)	17 (77.3)	0.104
Proximal muscle weakness, no. (%)	6 (50.0)	17 (77.3)	0.104
Comorbidities, no. (%)			
Arterial hypertension	1 (8.3)	2 (9.1)	0.941
Diabetes mellitus	2 (16.7)	1 (5.0)	0.273
malignancy	1 (8.3)	2 (9.1)	0.909
Laboratory examinations, median (IQR)			
CK, U/L	433 (66-2267)	269 (38-618)	0.769
LDH, U/L	384 (289-541)	320 (263-413)	0.592
Ferritin, μg/L	784 (285-1417)	494 (108-975)	0.235
ESR, mm/H	30 (23-44)	21 (19-32)	0.120
CRP, mg/L	8.29 (3.40-10.50)	3.41 (1.17-10.69)	0.377
Myositis-specific antibodies			
Anti-MDA-5 antibody, no. (%)	5 (41.7)	9 (40.9)	0.966
Anti-Jo-1 antibody, no. (%)	3 (25.0)	6 (27.3)	0.886
Anti-EJ antibody, no. (%)	1 (8.3)	3 (13.6)	0.999
Anti-PL-7 antibody, no. (%)	2 (16.7)	3 (13.6)	0.999
Anti-PL-12 antibody, no. (%)	1 (8.3)	1 (4.5)	0.999
HRCT patterns, no. (%)			
NSIP	7	19	0.109
OP	2	0	
NSIP+OP	2	2	
AIP	1	1	
Total HRCT score, mean (S.D.)	13.92 (3.66)	15.41 (5.35)	0.204
Ground-glass attenuation, mean (S.D.)	6.17 (3.35)	6.18 (3.35)	0.929
Airspace consolidation, mean (S.D.)	2.42 (2.71)	3.50 (3.29)	0.403
Interlobular septal thickening and/or reticular opacity, mean (S.D.)	4.33 (2.43)	4.45 (2.60)	0.986
Traction bronchiectasis, mean (S.D.)	1.00 (1.71)	1.27 (1.72)	0.606
Therapy modalities			
Initial Prednisolone dose (mg/day)	57 (30)	56 (31)	0.306
IVIg, no. (%)	5 (41.7)	8 (36.4)	0.761
PE, no. (%)	3 (25.0)	7 (31.8)	0.677

S.D., standard deviation; IQR, inter-quartile range**;** DM, dermatomyositis; PM, polymyositis; CADM, clinical asympmyopathic dermatomyositis; CK, creatine kinase; LDH, lactate dehydrogenase; ESR, erythrocyte sedimentation rate;CRP, C-reactive protein; anti–MDA-5, anti–melanoma differentiation–associated gene 5; HRCT, high-resolution computer tomography; NSIP, nonspecific interstitial pneumonia; OP, organizing pneumonia; AIP, acute pneumonia; IVIg, intravenous immunoglobulin; PE, plasma exchange.

**Figure 2:**
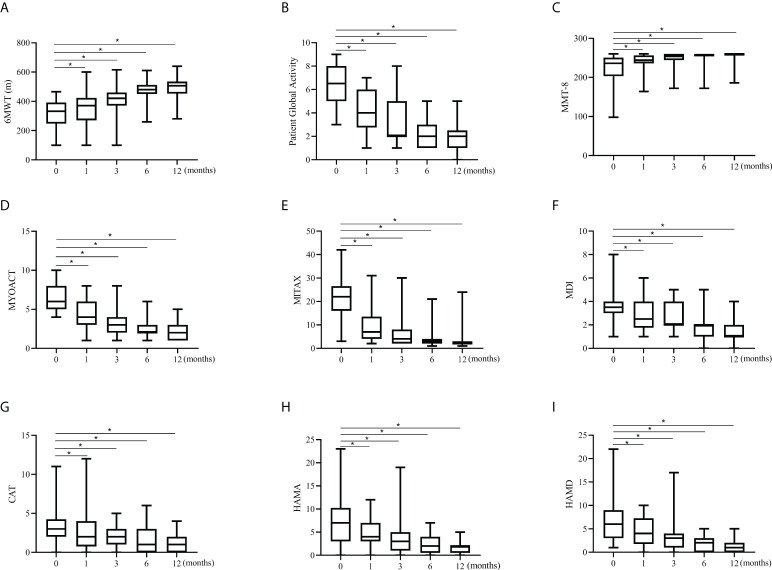
Changes in assessment scales in the prospective cohort. All the assessment scale indicators showed significant increases from baseline to 12 months in prospective investigation. Data are presented as box plots, where the boxes represent the 25th to 75th percentiles, the lines within the boxes represent the median, and the lines outside the boxes represent the minimum and maximum. *p<0.001, by paired Wilcoxon t-test, compared against baseline. **(A)** Changes in 6MWT. **(B)** Changes in Patient Global Activity. **(C)** Changes in MMT-8. **(D)** Changes in MYOACT. **(E)** Changes in MITAX. **(F)** Changes in MDI. **(G)** Changes in CAT. **(H)** Changes in HAMA. **(I)** Changes in HAMD. 6MWT, 6-min walking test; MMT-8, Manual Muscle Testing; MYOACT, Myositis Disease Activity Assessment visual analog scale; MITAX, Myositis Intention to Treat Activities Index; MDI, Myositis Damage Index; CAT, Cutaneous Assessment Tool; HAMA, Hamilton Anxiety Scale; HAMD, Hamilton Depression Scale.

**Figure 3:**
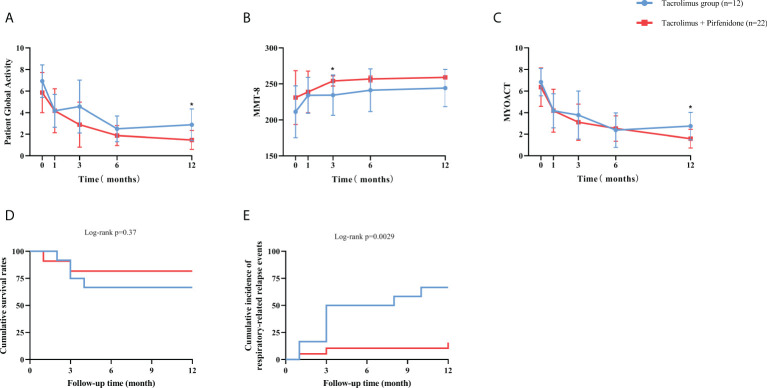
Therapeutic effect reflected by assessment scales and study endpoints between subgroups of prospective cohort. **(A)** The combination therapy group showed a significant improvement at 12 months than tacrolimus group in Patient Global Activity. **(B)** The combination therapy group showed a significant improvement at 3 months than tacrolimus group in MMT-8. **(C)** The combination therapy group showed a significant improvement at 12 months than tacrolimus group in MYOACT. **(D)** The combination therapy group had a significantly higher overall survival rate than tacrolimus group. **(E)** The tacrolimus group had a significantly higher respiratory-related relapse rate than combination therapy group. Blue line represents the tacrolimus group; Red line represents the combination therapy group. MMT-8: Manual Muscle Testing; MYOACT: Myositis Disease Activity Assessment visual analog scale. *p<0.05.

For the prospective subjects (n=34) as a whole, the treatment response to tacrolimus assessed by Myositis Assessment Scales was compared between baseline and each visit point. Significant improvements in cardio-pulmonary function, overall disease activity, muscular, respiratory, skin and mental involvements were observed compared to baseline (**Figure 2**). Daily prednisolone dose was significantly lower at last visit compared with the baseline (50 mg/d vs. 10 mg/d, data not shown). All the assessment scales showed progressive improvements after the initiation of tacrolimus treatment (**Figure 2**). We then further compared the efficacy and the assessment scales between patients with and without pirfenidone. Patients in combination therapy group showed a significant decrease in Patient Global Activity and MYOACT at 12 months and a significant improvement of MMT-8 at 3 months than those in control group (**Figures 3A** **–C**). Comparisons in terms of 6MWT, MITAX, MDI, CAT and mental assessment showed no difference between the two groups (**Supplementary Figure S4**). Kaplan-Meier curves showed no significant difference in cumulative survival rates between two groups (**Figures 3D, E** **)**. When compared with combination therapy group, tacrolimus group showed a significant higher respiratory-related relapse rates (p=0.0029).

**Figure 4:**
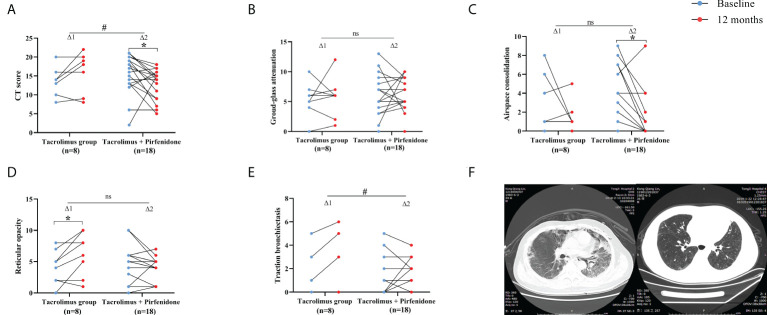
Therapeutic effect assessed by HRCT findings between subgroups of prospective cohort. **(A)** A significant reduction of CT score was seen in patients on combination therapy. The improvement was more significant in combination therapy group. **(B)** Non-significant improvements of ground-glass attenuation were seen in both groups. **(C)** A significant improvement of airspace consolidation was seen in patients on combination therapy. **(D)** A significant exacerbation of reticular opacity was seen in patients in tacrolimus group. **(E)** The improvement of traction bronchiectasia was more significant in combination therapy group. **(F)** Representative chest HRCT images of a patient at baseline and 12-month. △1 represents the change in the tacrolimus group; △2 represents the change in the combination therapy group. *p<0.05, compared against baseline. #p<0.05, comparison of changes in values between the two subgroups. ns means no significance.

The HRCT images were analyzed except for cases who died during observational period. 8 patients with tacrolimus monotherapy and 18 patients with combination therapy of tacrolimus plus pirfenidone were included for the analysis of the HRCT score, respectively. A significant improvement of total HRCT score was observed in combination therapy group (p=0.034), whereas no significant improvement was observed in tacrolimus group. In addition, the change of total HRCT score showed a significant difference between both groups (p=0.021) (**Figure 4A**). For combination therapy group, the extent of airspace consolidation improved significantly at 12th months compared to baseline values (p=0.009), although the delta value of two groups showed no statistically significant. Tacrolimus group showed a significant aggravation in reticular opacity when compared to baseline levels (p=0.045), but no significance of delta value was observed between two groups. Moreover, the combination therapy group showed a marked improvement as evaluated by changes in traction bronchiectasis when compared to tacrolimus group (p=0.016) (**Figures 4C** **–E**). Ground-glass attenuation was unchanged after treatment in both groups (**Figure 4B**). Representative chest HRCT images of an anti-MDA-5 antibody-positive patient were shown in **Figure 4F**. The patient experienced mediastinal emphysema and pneumothorax in left lung at baseline. A significant improvement in lung fibrosis lesions after “triple therapy” of 1 year was exhibited, with HRCT scores changed from 18 to 7 points.

No severe adverse events directly causing death were observed in our study. The incidence rate of drug-associated adverse events (AEs) was comparable between two groups. In both groups, opportunistic infections were the most commonly observed adverse events, accounting for 66.7% (8/12) and 45.5% (10/22) of patients in tacrolimus group and combination therapy group, respectively (**Supplementary Table 1**).

## Discussion

To our knowledge, this is the first prospective study to explore the efficacy of tacrolimus and GCs in combination with low-dose pirfenidone in IIM-ILD patients. First, we found that tacrolimus was superior in reducing the mortality rate and recurrence rate of IIM-ILD within the first year of treatment initial when compared with other conventional immunosuppressive agents in our retrospective study. In the prospective study, tacrolimus was confirmed as an effective and well-tolerated therapy in terms of improving muscle strength, ameliorating pulmonary dysfunction, and reducing disease activity. Although low -dose pirfenidone on top of tacrolimus has no impact on the survival of IIM-ILD patients, it may reduce the progression of pulmonary fibrosis and the rates of respiratory-related flare-ups in those patients. Our prospective study indicated that the treatment of tacrolimus on top of GCs followed by low-dose pirfenidone could improve both muscle and lung involvement of IIM-ILD patients, with a manageable safety profile. This “triple therapy” modality maybe an applicable treatment strategy for IIM patients with ILD.

It is well established that ILD is the gravest manifestation characterized by irreversible decline in lung function which ultimately results in a highly mortality. The prevalence of ILD is as high as 65% in IIM patients and considered as a dominant predictor for poor prognosis (25). The natural clinical course of ILD patients varies from slow progression to acute exacerbation, even death. Various dosages of GCs are considered as the mainstay of therapy. However, a majority of these patients were resistant to GCs monotherapy, and many of them experienced ILD deterioration or flare-ups during the process of GCs tapering (26). Higher dosages of GCs and immunosuppressive agents may provide more powerful immunosuppression and modify disease progression in a certain extent, but carry an increased risk of infections, osteoporosis, and any other adverse effects. Therefore, more cost-effective treatment modalities that could control progression of primary disease as well as improve pulmonary fibrosis are needed.

Th1-type pulmonary cells are significantly increased in GC-resistant PM/DM-ILD patients, hence, tacrolimus acting as a selectively suppressor of T lymphocytes proliferation may serve as an ideal treatment drugs (27). Several case reports and small-sample trails demonstrated that tacrolimus could improve disease-free survival and lung physiology in IIM-ILD patients (6, 28, 29). Similar findings were observed in our study that significant improvements of clinical manifestations and laboratory parameters result from tacrolimus have been seen in both the retrospective and prospective cohorts. In the retrospective cohort, we confirmed a preponderance of tacrolimus in improving survival rate and relapse rates of IIM-ILD patients comparing to other conventional immunosuppressive agents. We also found a remarkable reduction of GCs dosage after one-year treatment with tacrolimus in the prospective cohort, which is another possible advantage of tacrolimus. Ultimately, tacrolimus could not only improve muscle strength and overall disease activity but also permit a substantial spare of GCs.

Of note, our data provided further evidences supporting the efficacy and safety of low-dose pirfenidone in combination with tacrolimus therapy for IIM-ILD patients in the real world. Previous randomized clinical trials of pirfenidone demonstrated a slower decline of the lung function and sufficient drug tolerability in IPF patients (15, 16, 30). Furthermore, the potential benefits of pirfenidone were also seen in other types of pulmonary fibrosis, such as scleroderma-associated ILD (31) and clinically amyopathic dermatomyositis (17). More importantly, our study suggested that low-dose pirfenidone on top of tacrolimus had a lower respiratory-related recurrence rate as compared to those receive tacrolimus alone.

Chest HRCT is a reliable predictor of prognosis in IIM-ILD patients. A key finding in our study is that the “triple therapy” containing low-dose pirfenidone significantly improved the lung fibrosis according to the HRCT results from survived patients (26/34) in the prospective study. A significant decrease of total HRCT score was seen in the combination therapy group, which indicates that pirfenidone played a critical role in ameliorating pulmonary fibrosis for IIM-ILD patients. We observed that the airspace consolidation was decreased significantly after pirfenidone add-on treatment, which further confirmed the anti-inflammatory effect of pirfenidone in addition to the anti-fibrosis role. Reticular opacity got significantly worse in tacrolimus group, while no changes were seen in pirfenidone add-on group. In addition, patients in combination therapy groups showed a significant improvement of traction bronchiectasis compared to the control. These results suggested that low-dose of pirfenidone may have both anti-inflammatory and anti-fibrosis effects.

Semi-quantitative visual evaluation of HRCT scans can be challenging because of existence of some subtle lesions. Nevertheless, additional objective and quantitative assessment tools including serum biomarker and pulmonary function tests (PFTs) should be introduced to monitoring disease progression. PFTs are recognized as a useful and non-invasive measurement of lung function. However, the results of PETs were not analyzed in our study because up to 32.3% of the baseline data were unavailable owing to the severity of the respiratory failure of these patients. In addition, due to the rapid COVID-19 pandemic, most of patient in our center were unable to perform PFTs as proposed. Based on the limited data in our study, no additional PFTs improvement was observed in those received pirfenidone compared to the controls.

The common drug-associated adverse effects in our study are infections, disturbances in glucose metabolism, and electrolyte imbalance. Opportunistic infections, such as pneumocystis carinii, CMV and EBV activation, were frequently observed in patients who received combined immunosuppressive treatments, and these infections often trigger original disease flare-ups and exacerbation of ILD (32). In our study, exacerbation of ILD was the dominant cause of death. Those patients either experienced rapid progression of ILD or opportunistic infections secondary to exacerbation of ILD or excessive immunosuppressive therapies. It is of great significance to prevent potential infections and other complications in addition to control the progression of lung fibrosis in IIM-ILD patients. Thus, trimethoprim/sulfamethoxazole (TMP/SMX) should be administrated to prevent pneumocystis jiroveci pneumonia (PCP). Furthermore, monitoring the serum CMV and EBV levels are needed in order to make timely interventions. It is important to prevent potential infection and other complications in patients, but it is also equally important to give appropriate treatment of progressive lung disease. As reported, the most common adverse events of pirfenidone were gastrointestinal-related nausea and dyspepsia, which are generally responsive to dosage reduction (15, 16, 30). High-dose pirfenidone may also reduce the compliance of patients due to the financial burden. To some extent, the use of pirfenidone could be limited by side effects as well as treatment costs. Consequently, administration of low-dose pirfenidone may be a rational alternative for IIM-ILD patients. In our study, all patients responded and tolerated well to this “triple therapy”, with manageable side effects.

Because IIM- ILD is a life-threatening condition, it is difficult to conduct randomized clinical trials. There are several limitations in our study. First, this is a single-center, open-label trial, which may have potential bias. Second, information bias and missing data were inevitable in the retrospective study due to the retrospective nature. Third, the limited sample size and relatively short follow-up period was also an issue, especially in prospective study. Forth, not all patients have data on PFTs in the prospective study either due to the severity of the respiratory failure at baseline or COVID-19 epidemic. Last, all patients in the present study were Chinese and it is unclear whether these findings will apply to individuals of different ethnicities. Therefore, further multi-center, randomized control trials with larger population are needed to confirm our results. The long-term outcome and mortality is also warranted to analyze.

In summary, this study demonstrated that IIM-ILD patients treated with tacrolimus showed significant improvements in mortality and flare-ups when compared to other immunosuppressive agents. Tacrolimus results in multidimensional improvements in both myositis and pulmonary involvement, which could serve as a promising therapeutic alternative in the management of IIM-ILD. One year of “triple therapy” not only slowed the pulmonary fibrosis progression but also reduced respiratory-related flare-ups in IIM-ILD patients. This “triple therapy” seems to be well tolerated and should be considered in the future treatment of IIM-ILD patients. Nevertheless, this conclusion should be confirmed by further large-sample, randomized controlled studies.

## Data availability statement

The original contributions presented in the study are included in the article/**Supplementary Material**. Further inquiries can be directed to the corresponding authors.

## Ethics statement

The studies involving human participants were reviewed and approved by Institutional Review Board of Tongji Medical College, Huazhong University of Science and Technology in accordance with the principles of the Declaration of Helsinki and registered online at the Chinese clinical trial (ChiCTR2100043595). The patients/participants provided their written informed consent to participate in this study. Written informed consent was obtained from the individual(s) for the publication of any potentially identifiable images or data included in this article.

## Author contributions

All authors were involved in drafting the article or revising it critically for important intellectual content, and all authors approved the final version to be published. Study conception and design. LD and JZ. Acquisition of data. YC, ZB, and ZZ. Analysis and interpretation of data. YC, ZB, and QH.

## Funding

This work was supported by grants from National Natural Science Foundation of China (81771754 and 81974254), Scientific and Technological Innovation Projects of Hubei Province (2021CFB058), Tongji Hospital Clinical Research Flagship Program (2019CR206), and Science Foundation of Tongji Hospital (2018A09).

## Acknowledgments

The authors would like to thank all patients participated in this study.

## Conflict of interest

The authors declare that the research was conducted in the absence of any commercial or financial relationships that could be construed as a potential conflict of interest.

## Publisher’s note

All claims expressed in this article are solely those of the authors and do not necessarily represent those of their affiliated organizations, or those of the publisher, the editors and the reviewers. Any product that may be evaluated in this article, or claim that may be made by its manufacturer, is not guaranteed or endorsed by the publisher.

## Supplementary material

Supplementary Figure 1 The flowchart of patients included in the retrospective cohort.

Supplementary Figure 2 Kaplan-Meier curves of the study outcomes used for comparison between the Tacrolimus and other common immunosuppressant including cyclophosphamide, methotrexate and azathioprine. CTX: cyclophosphamide; MTX: methotrexate; AZA: azathioprine.

Supplementary Figure 3 The flowchart of patients included in the prospective cohort.

Supplementary Figure 4 Therapeutic effect reflected by assessment scales and study endpoints between subgroups of prospective cohort. **(A)** The comparison of changes in 6MWT between subgroups. **(B)** The comparison of changes in MITAX between subgroups. **(C)** The comparison of changes in MDI between subgroups. **(D)** The comparison of changes in CAT between subgroups. **(E)** The comparison of changes in HAMA between subgroups. **(F)** The comparison of changes in HAMD between subgroups. P was evaluated by Log-rank test with Bonferroni adjustment. 6MWT, 6-min walking test; MITAX, Myositis Intention to Treat Activities Index; MDI, Myositis Damage Index; CAT, Cutaneous Assessment Tool; HAMD, Hamilton Depression Scale.
