# Supplementary material for: The efficacy and safety of tacrolimus on top of glucocorticoids in the management of IIM-ILD: A retrospective and prospective study
Source: Front Immunol. 2022 Sep 2;13:978429. doi: 10.3389/fimmu.2022.978429 (PMC9479328; doi:10.3389/fimmu.2022.978429)
Supplement: Supplementary file 1 [file Table_1.docx]

Supplementary table 1. Adverse events during immunosuppressive treatment, no. of patients (%).

|  | Tacrolimus group  (n=12) | Tacrolimus + Pirfenidone group (n=22) |
| --- | --- | --- |
| Infection (total) | 8 (66.7) | 10 (45.5) |
| Bacterial infection | 3 (41.7) | 1 (4.5) |
| Mycoplasma infection | 2 (16.7) | 0 |
| CMV | 1 (8.3) | 1 (4.5) |
| HSV | 1 (8.3) | 2 (9.1) |
| EBV | 1 (8.3) | 2 (9.1) |
| PCP | 3 (41.7) | 3 (13.6) |
| Candidiasis | 0 | 1 (4.5) |
| Hyperlipemia | 1 (8.3) | 1 (4.5) |
| Diabetes mellitus | 1 (8.3) | 3 (13.6) |
| Hyperglycemia | 3 (41.7) | 1 (4.5) |
| Hyponatremia | 1 (8.3) | 0 |
| Hypokalemia | 1 (8.3) | 1 (4.5) |
| Hyperkalemia | 0 | 1 (4.5) |
| Fremitus | 1 (8.3) | 0 |

Abbreviations: CMV: cytomegalovirus; HSV: herpes simplex virus; EBV: Epstein-Barr virus; PCP: pneumocystis pneumonia.
