# Supplementary figures and images for: The efficacy and safety of tacrolimus on top of glucocorticoids in the management of IIM-ILD: A retrospective and prospective study
Source: Front Immunol. 2022 Sep 2;13:978429. doi: 10.3389/fimmu.2022.978429 (PMC9479328; doi:10.3389/fimmu.2022.978429)

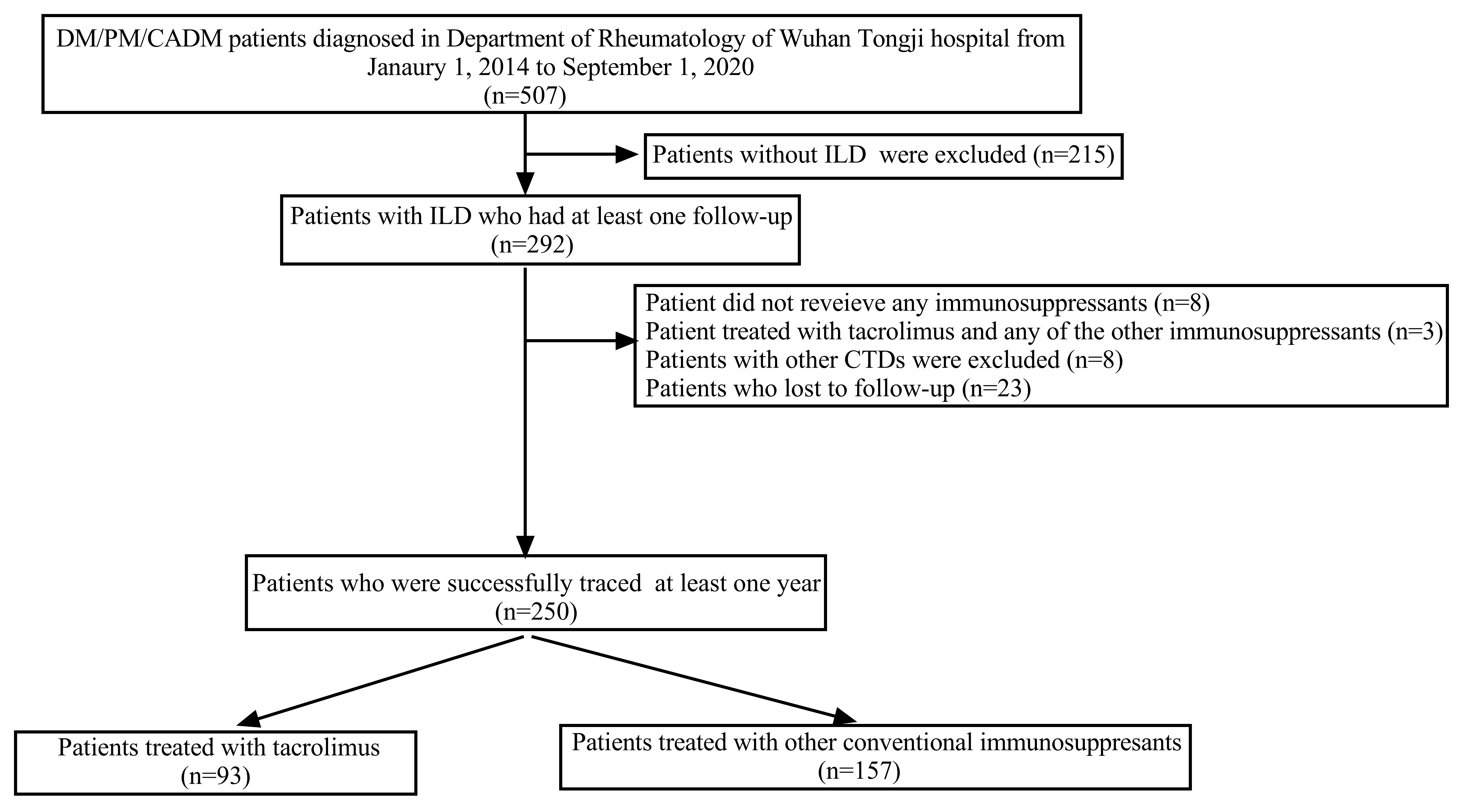

Supplement: Supplementary Figure 1 — The flowchart of patients included in the retrospective cohort. [file Image_1.tif]

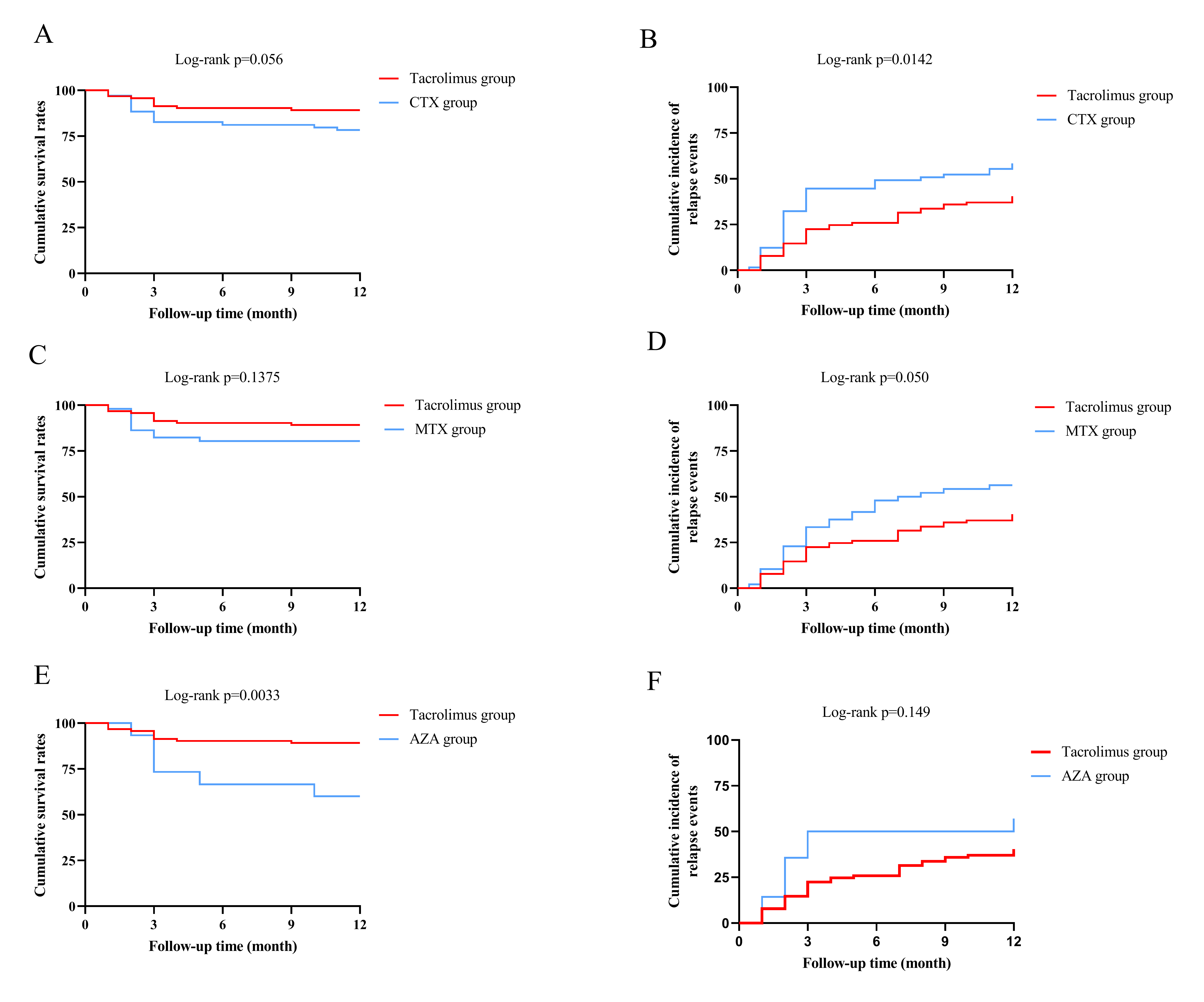

Supplement: Supplementary Figure 2 — Kaplan-Meier curves of the study outcomes used for comparison between the Tacrolimus and other common immunosuppressant including cyclophosphamide, methotrexate and azathioprine. CTX: cyclophosphamide; MTX: methotrexate; AZA: azathioprine. [file Image_2.tif]

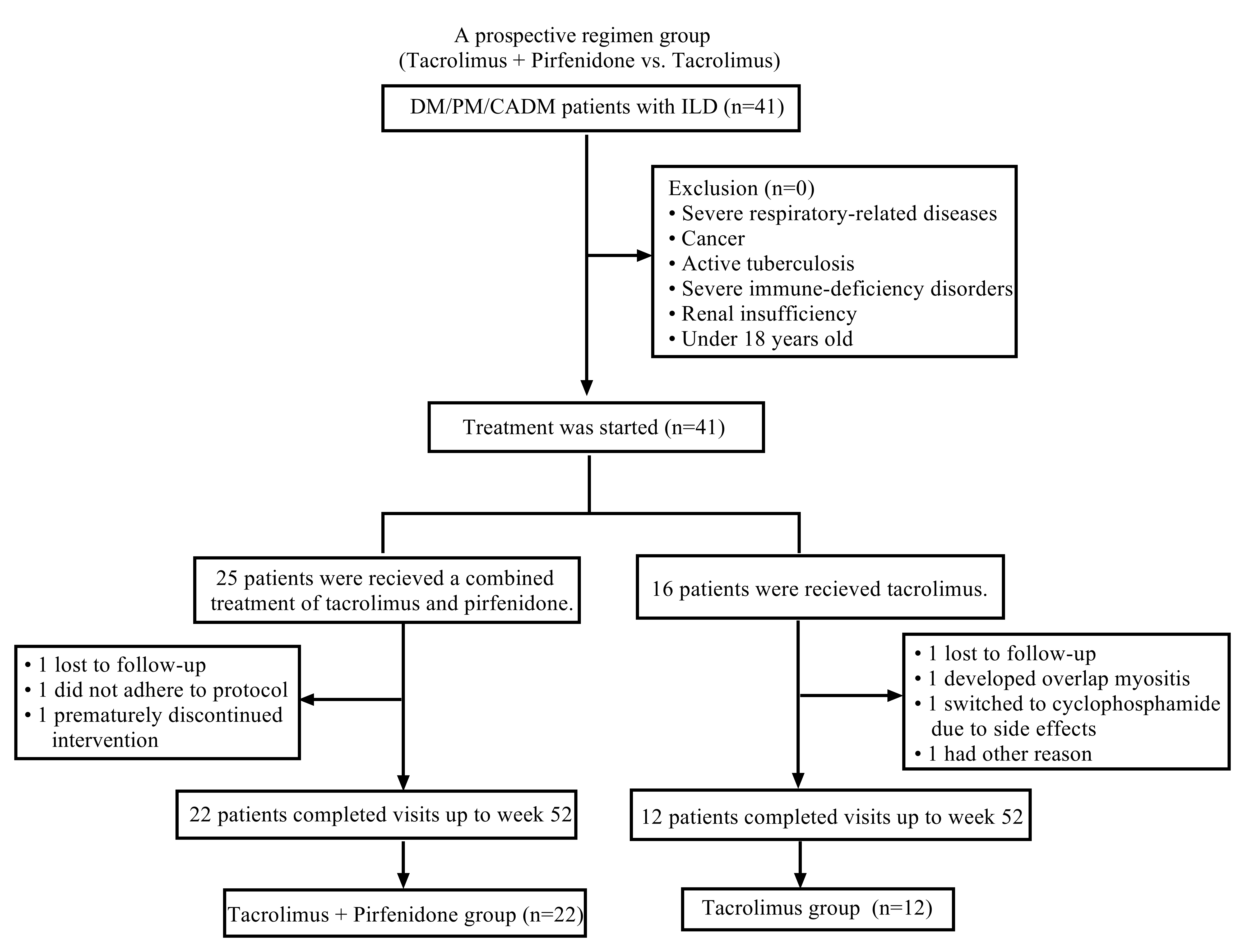

Supplement: Supplementary Figure 3 — The flowchart of patients included in the prospective cohort. [file Image_3.tif]

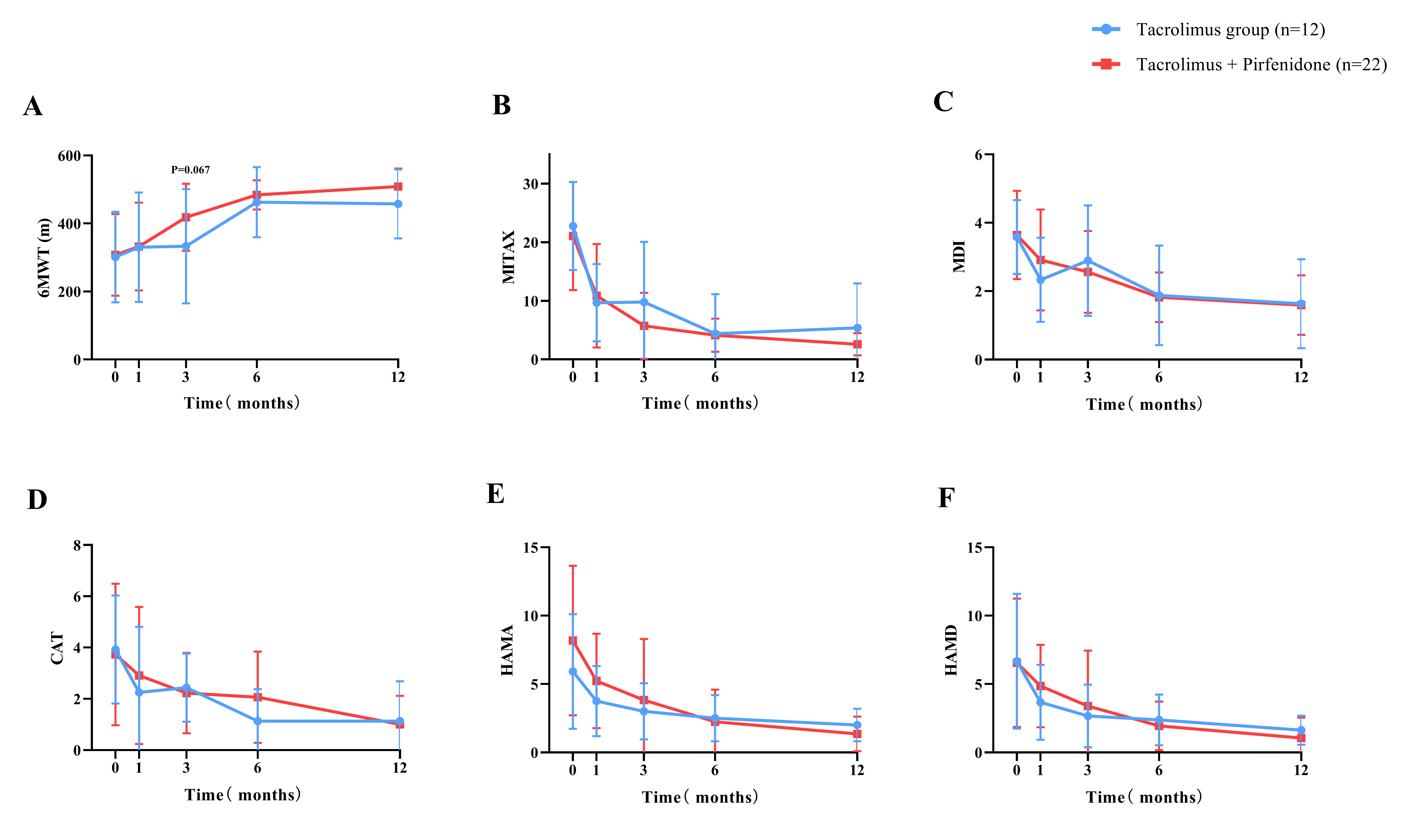

Supplement: Supplementary Figure 4 — Therapeutic effect reflected by assessment scales and study endpoints between subgroups of prospective cohort. (A) The comparison of changes in 6MWT between subgroups. (B) The comparison of changes in MITAX between subgroups. (C) The comparison of changes in MDI between subgroups. (D) The comparison of changes in CAT between subgroups. (E) The comparison of changes in HAMA between subgroups. (F) The comparison of changes in HAMD between subgroups. P was evaluated by Log-rank test with Bonferroni adjustment. 6MWT, 6-min walking test; MITAX, Myositis Intention to Treat Activities Index; MDI, Myositis Damage Index; CAT, Cutaneous Assessment Tool; HAMD, Hamilton Depression Scale. [file Image_4.tif]
